# Combined impairments in vision, hearing and cognition are associated with greater levels of functional and communication difficulties than cognitive impairment alone: Analysis of interRAI data for home care and long-term care recipients in Ontario

**DOI:** 10.1371/journal.pone.0192971

**Published:** 2018-02-15

**Authors:** Dawn M. Guthrie, Jacob G. S. Davidson, Nicole Williams, Jennifer Campos, Kathleen Hunter, Paul Mick, Joseph B. Orange, M. Kathleen Pichora-Fuller, Natalie A. Phillips, Marie Y. Savundranayagam, Walter Wittich

**Affiliations:** 1 Department of Kinesiology & Physical Education, Wilfrid Laurier University, Waterloo, Ontario, Canada; 2 Department of Health Sciences, Wilfrid Laurier University, Waterloo, Ontario, Canada; 3 Toronto Rehabilitation Institute – University Health Network, Toronto, Ontario, Canada; 4 Department of Psychology, University of Toronto, Toronto, Ontario, Canada; 5 Faculty of Nursing, University of Alberta, Edmonton, Alberta, Canada; 6 Faculty of Medicine/Division of Geriatric Medicine, University of Alberta, Edmonton, Alberta, Canada; 7 Department of Surgery, Faculty of Medicine, University of British Columbia, Vancouver, British Columbia, Canada; 8 School of Communication Sciences and Disorders, Western University, London, Ontario, Canada; 9 Department of Psychology, University of Toronto, Mississauga, Ontario, Canada; 10 Department of Psychology, Concordia University, Montreal, Quebec, Canada; 11 School of Health Studies, Western University, London, Ontario, Canada; 12 School of Optometry, University of Montreal, Montreal, Quebec, Canada; 13 CRIR/MAB-Mckay Rehabilitation Centre of West-Central Montreal Health, Montreal, Quebec, Canada; 14 CRIR/Institut Nazareth et Louis-Braille du CISSS de la Montérégie-Centre, Montreal, Quebec, Canada; West Chester University of Pennsylvania, UNITED STATES

## Abstract

**Objectives:**

The objective of the current study was to understand the added effects of having a sensory impairment (vision and/or hearing impairment) in combination with cognitive impairment with respect to health-related outcomes among older adults (65+ years old) receiving home care or residing in a long-term care (LTC) facility in Ontario, Canada.

**Methods:**

Cross-sectional analyses were conducted using existing data collected with one of two interRAI assessments, one for home care (n = 291,824) and one for LTC (n = 110,578). Items in the assessments were used to identify clients with single sensory impairments (e.g., vision only [VI], hearing only [HI]), dual sensory impairment (DSI; i.e., vision and hearing) and those with cognitive impairment (CI). We defined seven mutually exclusive groups based on the presence of single or combined impairments.

**Results:**

The rate of people having all three impairments (i.e., CI+DSI) was 21.3% in home care and 29.2% in LTC. Across the seven groups, individuals with all three impairments were the most likely to report loneliness, to have a reduction in social engagement, and to experience reduced independence in their activities of daily living (ADLs) and instrumental ADLs (IADLs). Communication challenges were highly prevalent in this group, at 38.0% in home care and 49.2% in LTC. In both care settings, communication difficulties were more common in the CI+DSI group versus the CI-alone group.

**Conclusions:**

The presence of combined sensory and cognitive impairments is high among older adults in these two care settings and having all three impairments is associated with higher rates of negative outcomes than the rates for those having CI alone. There is a rising imperative for all health care professionals to recognize the potential presence of hearing, vision and cognitive impairments in those for whom they provide care, to ensure that basic screening occurs and to use those results to inform care plans.

## Introduction

Sensory (vision and hearing) and cognitive impairments are highly prevalent among older adults (65+ years old) and are associated with difficulties in multiple domains including communication, mood, functional ability, and social engagement. The literature has focused on the influence of a single impairment (e.g., hearing impairment, vision impairment, or cognitive impairment) and has infrequently considered their combined influence on health and well-being in older adults. Researchers have even less frequently investigated these combined effects among older adults receiving continuing care services (e.g., home care or long-term care).

Sensory and cognitive impairments are highly prevalent among older adults and increase with age.[1] The prevalence is expected to increase over the coming decades due mainly to population aging as a result of various factors including improved health care delivery, efforts in health prevention and improved nutrition, to name a few. Hearing impairment is the third most prevalent chronic condition among older adults in the United States[2]. Approximately 65% of Canadians aged 70 years and older have a hearing impairment, with both incidence and prevalence rates rising with each decade of life.[3] Today, 6.5 million Americans over the age of 65 years have a severe visual impairment and experts predict that by 2030 rates of severe vision impairment will double along in the country’s aging population.[4] Blindness or low vision affects approximately 1 in 28 Americans older than 40 years.[5] Within the next two decades, it is anticipated that both age-related hearing impairment (HI) and vision impairment (VI) will rank within the top 10 burdens of disease among those living in middle- and high-income countries while cognitive impairment, including Alzheimer’s dementia (AD) and other forms of dementia, are anticipated to be in the top four.[6]

### Consequences of age-related hearing impairment, vision impairment and dual sensory impairment

Each of HI and VI is associated with adverse outcomes. For example, HI is associated with poor self-rated health,[7] difficulties with activities of daily living (ADLs; e.g., eating, bathing, dressing) and instrumental ADLs (IADLs; e.g., using the telephone, managing finances),[7–10] difficulty with memory,[7] frailty and falls. [11].

Similarly, VI has been linked to multiple adverse outcomes including an increased risk of mortality, [10, 12] difficulties with independence in activities of ADLs and IADLs,[10, 13] difficulty with mobility [14] and reduced social participation.[13, 15] Individuals with VI also are more likely than those without VI to receive community-based supports (e.g., home care, meals-on-wheels).[14]

Dual sensory impairment (DSI) involves a combination of both HI and VI and is a unique disability whereby persons cannot accommodate for the loss in one sense by using the other sense. DSI profoundly influences individuals’ abilities to gather information about their surroundings and to communicate with others. DSI also restricts their capacity to participate fully in a range of social environments without some means of assistance or support (e.g., sign-language interpreter, communication specialist).[16] Estimates of the prevalence of DSI in North America typically range from 3% to 21%,[17, 18] are between 6 to 7% in several European countries,[19–22], generally increase with age[17], and are higher among those receiving home care services or residing in a long-term care facility.[13, 23]

A recent systematic review provides clear evidence that older people with DSI are a particularly vulnerable group.[24] Older persons with DSI experience difficulties performing ADLs and IADLs,[10, 18, 25, 26] are at increased risk for depression [27, 28] and mortality,[10] have significantly impaired communication function and social isolation,[29] show impaired mobility,[26] and when asked, often express concerns about the future.[26] Despite those with DSI being at great risk for multiple adverse events, there is very limited research on older adults with DSI in Canada.[23, 27, 30] Understanding the prevalence and functional consequences of acquired DSI with advanced age is important given that the population of older adults is rapidly growing and age-related sensory decline is common.

### Relationship between sensory and cognitive impairments

It is well documented that AD and other forms of dementia are associated with progressive declines in ADLs, IADLs, cognition, behavior, visuospatial skills, mobility, overall quality of life, higher rates of depression, caregiver burden and institutionalization.[31, 32] Importantly, recent evidence suggests that associations exist between sensory and cognitive impairments[33] and that the functional consequences of experiencing both may be compounded. For instance, studies have shown that age-related HI is linked to an increased risk of cognitive decline and incident dementia,[34–36] including evidence from imaging studies showing that individuals with HI have higher rates of brain atrophy in the right temporal lobe and reductions in total brain volume, compared to individuals without HI.[37] Similarly, a large population-based study of older adults in the United States found an increased odds of poor cognitive function among individuals with vision impairment.[38] A recent meta-analysis estimated that the proportion of dementia cases potentially attributable to hearing loss (i.e., the population attributable fraction) was, at 9%, higher than for any other non-genetic risk factor. This was likely due to the high prevalence of age-related hearing loss and large effect estimates in previous longitudinal studies that examined associations between hearing loss and incident dementia.[39]

Several studies have examined both single- and dual-sensory impairment and cognitive impairments among older adults, and how they act together to influence cognitive, emotional and physical health. Of these, four studies reported a significant association between sensory impairments (HI, VI or DSI) and CI. However, in several of these studies, the influence of sensory impairments was less pronounced, or not significant, after adjusting for multiple potential confounding variables,[40–43] and one did not find an important relationship.[44] Another study found an increased likelihood of functional disability and poor self-rated health among those with combinations of sensory and cognitive impairments. The highest odds ratios, regardless of outcome, were among those with combined dual sensory and cognitive challenges.[45] Only one of these studies targeted older adults who either required health care assistance in the community or were eligible for long-term care (LTC) insurance,[41] with the other four focusing on otherwise healthy older adults.

There is clearly a need to better understand the associations between health-related outcomes and sensory/cognitive impairment (alone and in combination) among individuals receiving home care or residing in a LTC facility given the number of care recipients and the resources being allocated to these services. For example, in Ontario alone, public funding for home care costs roughly $2.4 billion annually [46, 47] and $3.97 billion for LTC [48] and yet very little is known about the interplay between sensory and cognitive impairment in these two care settings and the potential cost/benefit advantages of addressing sensory issues.

The objective of the current study was to understand the potentially compounded effects of sensory (i.e., HI and VI) and cognitive impairments on a series of health-related outcomes in two cohorts of older adults in Ontario receiving ongoing health care either in the community or in a residential setting. Given the size and complexity of the cohorts, the scope of questions to be addressed, and the exploratory nature of this novel study, our goal in this paper is to provide the necessary descriptive and prevalence-based information that will serve as the foundation for future publications.

We began by creating a detailed profile of the individuals in these two cohorts to characterize their current levels of functioning and their associations with sensory and cognitive status. Given the volume of items available at the individual level within each cohort (roughly 300 items), we chose to focus our analyses on several key domains (e.g., functional ability, cognitive performance, communication, mood/behaviour, physical health). We then focused on understanding the additional consequences of cognitive impairment on a person’s functional and health status beyond the challenges attributable to the presence of sensory impairments alone. For example, we considered whether there were meaningful differences between those with cognitive impairment alone versus those with cognitive impairment and single or dual sensory impairments.

## Materials and methods

### Design

The current study was a cross-sectional analysis of secondary data collected in Ontario using the Resident Assessment Instrument for Home Care (RAI-HC) and the Minimum Data Set 2.0 (MDS 2.0) for LTC. The RAI-HC and the MDS 2.0 were created by interRAI (www.interrai.org), a not-for-profit organization of researchers and clinicians representing over 30 different countries. The instruments are standardized clinical assessments used primarily for clinical decision-making based on domains such as sensory status and communication, cognitive and behavioral patterns, psychosocial well-being, informal and formal support services, physical functioning, and medical diagnoses.[49] The majority of items in these two assessments (for home care and LTC) are either very similar or identical in both the wording of the items and the response options.

The RAI-HC assessment is mandated in Ontario for all long-stay home care clients (i.e., those expecting to receive services for at least 60 days). The MDS 2.0 is mandated for all LTC residents in the province. As such, both instruments are used in Ontario as part of standard clinical practice. Trained care coordinators (typically registered nurses) complete the assessments using many sources of information, including interactions with the care recipient, informal caregivers, health providers (e.g., primary care physician), and information from clinical records. The typical reassessment interval for the RAI-HC is every six to twelve months, unless there is a change in the individual’s clinical status. The MDS 2.0 is administered in full upon admission into a LTC facility and yearly thereafter. A shorter version of the MDS 2.0 is completed on a quarterly basis. The completed assessments are submitted to the Canadian Institute for Health Information (CIHI, www.cihi.ca), which stores the data in a national data warehouse. Researchers can apply to CIHI to receive de-identified data for research purposes.

### Sample

The data were the most recent available from CIHI for Ontario when the analysis began in early 2015. The sample for our analyses included a cohort of 291,824 unique home care clients and a second cohort of 110,578 unique LTC residents, 65 years of age and older, residing in Ontario, who had an interRAI assessment completed between 2009 and 2014. For those individuals who had multiple assessments, we analyzed the most recent assessment. This project was reviewed and approved by the research ethics board at Wilfrid Laurier University (REB #4184).

### Measures used for classification of sensory and cognitive impairment

The primary focus of this paper was on the independent and combined influences of sensory impairments (i.e., HI, VI, DSI) and cognitive impairment (CI) on relevant aspects of physical, social and emotional functioning. We defined seven mutually exclusive groups of individuals with single or combined impairments (i.e., HI alone, VI alone, DSI, HI+CI, VI+CI, DSI+CI, CI alone) and an eighth group of individuals who had none of these impairments. If an individual had both HI and CI then they could not populate both the combined impairment group (CI+ HI) *and* the single impairment group (just HI or CI). Instead, the individual was classified only as having a combined impairment. This also was true for DSI, such that a person with both HI and VI was *not* classified as having HI alone nor as having VI alone, but rather only as having DSI.

Sensory impairment was defined and determined using the items in the RAI-HC and MDS 2.0 instruments. Ratings of hearing and vision function were assigned scores by a trained health care professional based on an interview with the individual while they were using any corrective hearing or vision devices that the person would typically use (e.g., hearing aid, glasses). The possible functional hearing performance ratings range from 0 to 3. A rating of 0 represents adequate hearing, 1 equals mildly impaired hearing (e.g., individual has difficulty in situations other than in a quiet setting), 2 indicates moderately impaired hearing (e.g., a talker has to alter speech tone/quality/loudness to be understood), and 3 indicates severely impaired hearing function (i.e., no apparent useful hearing). The HI criterion correlates with the Hearing Handicap Inventory screener,[50] indicating that it is a valid measure of a person’s perceptions of the effects of HI on their emotional well-being and their capacity for everyday activities (e.g., their ability to communicate with others, use the telephone, etc.). The possible ratings of functional vision range from 0 to 4, with 0 indicating adequate vision, 1 mildly impaired vision (e.g., difficulty reading regular print in newspapers/books), 2 moderately impaired vision (e.g., unable to see newspaper headlines, but can identify objects), 3 highly impaired vision (e.g., object identification is in question, but the individual appears to follow objects with their eyes), and 4 severely impaired vision function (e.g., individual sees only lights, colours, or shapes, but has no useful vision, even with the use of their assistive devices/strategies). Impairment in either of these senses was defined as a score of 1 or greater, indicating at least mild HI or VI.

The presence of DSI was determined using an existing scale embedded within the interRAI tools, the Deafblind Severity Index (DbSI).[51] The DbSI combines the functional hearing and vision items described above, to create a five-point scale (0 for no impairment in either sense to 5 for severe impairment in both senses). A score of three or higher on the DbSI was used to identify individuals with DSI since it represents the presence of at least mild impairment in both vision and hearing. Previous research provides preliminary evidence of concurrent validity insofar as an increasing score on the DbSI corresponds with both greater difficulty in performing IADLs and greater difficulty interacting with others[51]. The sensory items used in the DbSI also each show excellent test-retest reliability (hearing: kappa = 0.83; vision: kappa = 0.85).[51]

The presence of CI was determined and defined using the Cognitive Performance Scale (CPS), which is a hierarchical scale including four items pertaining to short-term memory, independence in eating, expressive communication, and decision-making. The individual items are scored in a variety of ways. For example, both expressive communication and capacity for decision-making are scored from 0 to 4, with higher scores indicating a greater level of difficulty. The item for eating is scored from 0 to 8, with higher scores representing an increasing need for assistance. Short-term memory is a dichotomous variable scored to represent the presence of short-term memory problems (1 = yes, 0 = no). The items are combined in a hierarchical fashion to create the CPS score, which ranges from 0 (intact) to six (very severely impaired). The CPS has been validated against the Mini-Mental State Examination (MMSE), the Test for Severe Impairments,[52] and the Montreal Cognitive Assessment (MoCA). A score of 2 or greater on the CPS was used to identify and classify individuals as having CI and corresponds to mild/moderate impairment on the MMSE (average score of 23.8/30) or the MoCA (average score of 20.1/30).[53] Individuals rated as having no sensory or cognitive impairment were included for comparison purposes (i.e., “no impairment” group).

### Measures of physical, social and emotional functioning

Given the number of items within the assessment tools, we chose to elaborate more fully in the text on those items that are less easy to discern based on the data provided in the tables. A full description of each item and how they are coded is available from the corresponding author (DMG).

#### Communication

Two items on the RAI-HC and MDS 2.0 capture communication ability. One item captures expressive communication and is also included in the CPS scale, which was used to define the presence of CI. As such, when we discuss the results related to communication difficulties among those with CI or any combination of CI plus a sensory impairment, we do not report the results for expressive communication because doing so would inflate the values. However, the results are reported for those who do not have CI alone or in combination with sensory impairment(s). Expressive communication refers to the ability to make oneself understood by others and the item was scored from 0 (always understood) to 4 (rarely or never understood). The other communication item captures receptive communication (ability to understand) and also is scored from 0 to 4. Scores higher than 2 on both communication items was rare in the current sample (typically less than 10% of cases). We re-coded each item to create two new variables to be used in the analysis, each of which was coded as follows: no communication difficulty (individuals with a score of 0 on the original variable), mild degree of difficulty (individuals with a score of 1), and moderate-to-severe difficulty (individuals with scores of 2, 3, or 4).

In addition to the items described previously, a number of health index scales also can be generated to assess clinical status using items embedded within the RAI-HC and MDS 2.0. Since the assessments are completed electronically, the scales can be automatically generated by the software and can assist professionals in developing an individualized care plan. Here we selected five health index scales to include in the analysis because of their relationship with sensory and/or cognitive impairment(s). Each of these scales is described in detail below.

#### Depression

The Depression Rating Scale (DRS) is a summative scale across seven items measuring an individual’s mood and behaviour patterns (e.g., negative statements, persistent anger, expression of unrealistic fears, repetitive health complaints, repetitive anxious complaints, sad/pained/worried facial expressions, and tearfulness). Each of the seven items is scored from 0 to 2 and then summed to create a score on the DRS ranging from 0 to 14. This scale has been validated against the Hamilton Depression Rating Scale and the Cornell Scale for Depression[54] and has good convergent/divergent validity and acceptable reliability in older palliative home care clients.[55] A cut-point of 3 or greater was used for the DRS as this has been shown to be a valid indicator for a clinical diagnosis of depression.[56]

#### Activities of daily living (ADLs)

The ADL Self-performance Hierarchy Scale (ADL-SHS)[57] measures functional ability on a seven-point scale (0 for independent to 6 for total dependence) by capturing the process of ADL disablement across four items. The composite score gives higher weightings to “late loss” ADLs, which represent activities that individuals can often do own their own, or with a limited degree of assistance, until later in their life (e.g., eating), compared to middle loss ADLs (e.g., locomotion, toileting) or early loss ADLs (e.g., personal hygiene), which represent the first activities with which individuals require assistance. The ADL-SHS has been validated against the Barthel Index.[58] For the purposes of this study, a score of 2 or higher on the ADL-SHS (the point at which an individual can no longer complete all of their ADLs independently) was used to define ADL impairment [49, 57] as has been done in previous research.[23, 59]

#### Instrumental ADLs (IADLs)

The IADL Involvement Scale is a summative scale generated from seven items within the RAI-HC, including the activities of meal preparation, ordinary housework, managing finances, managing medications, phone use, shopping, and transportation. Since they are not applicable to those living in LTC, they are not included on the MDS 2.0 assessment. The item scoring ranges from 0 (independent) to 3 (performed by others) on each activity, yielding a score ranging from 0 to 21. A higher score indicates a greater level of impaired function in performing these tasks and is correlated with the Lawton Index.[60] For the purposes of this study, a cut-point of 14 or higher indicates moderate/major difficulty in performing IADLs (i.e., clients who were unable to complete the majority of IADLs independently scored in this range).

#### Pain

The Pain Scale[61] uses two items measuring the frequency and intensity of pain to create a four-point scale (0 for no pain to 3 for severe daily pain). The scale has established criterion validity when compared with the ten-point Visual Analog Scale (VAS).[61] A cut-point of 2 or higher was chosen since this represents the transition from periodic pain, to daily or severe daily pain.

#### Health instability

The Changes in Health, End-Stage Disease, Signs and Symptoms (CHESS)[62] scale identifies individuals who are at risk for health instability based on the presence of six health symptoms: vomiting, dehydration, leaving food uneaten, weight loss, shortness of breath, and edema. These conditions are scored as 0 (no symptoms), 1 (single symptom), or 2 (more than one symptom). The score for the six health symptoms is combined with the individual’s score on three other items measuring end-stage disease, decline in cognition, and ADL decline (0 for not present, or 1 for present for each of these three additional items). The combined score results in a six-point scale that ranges from 0 (no health instability) to 5 (highest level of health instability). The CHESS Scale is a significant predictor of mortality and there is a 60% increase in mortality with each single-point increase on the scale.[63] A cut-point of 2 or higher was used to determine health instability based on previous research showing a marked increase in the hazard ratio for mortality among home care clients scoring 2 or higher compared with those scoring zero or one on the CHESS.[64]

### Analysis

The data used for analysis constitute a near census of home care clients and LTC residents in Ontario. Given the large sample sizes in the two cohorts, we have chosen not to conduct statistical tests to examine differences between the eight subgroups of interest since even the most minimal absolute differences would result in a statistically significant finding that in all likelihood would not be meaningful in practice. Instead, we chose to report proportion values to highlight differences in rates across the seven groups representing the combinations of sensory and cognitive impairment (i.e., HI, VI, DSI, CI, CI+HI, CI+VI, CI+DSI) and the eighth group representing no sensory and no cognitive impairment. The seven subgroups were compared, within each cohort (i.e., within home care and within LTC), across various items included in the assessments, both to understand the key results within a given cohort as well as any differences between the two care settings. Given the large number of variables that were possible to include in the analysis (roughly 300 items on each assessment), we have highlighted key results here and have included a supplementary document with additional findings for each cohort (Tables A and B in S1 File).

Age can increase the risk of certain negative health issues (e.g., impaired functional ability, health instability) and is a risk factor for both sensory and cognitive impairment.[17] In order to better understand the influence of age in general versus the contribution of sensory/cognitive impairments specifically, a stratified analysis was conducted. Age was recoded into three categories (65–74, 75–84, and 85+ years). The relationship between the seven combinations of sensory and cognitive impairments was compared with several health-related and functional outcomes (e.g., ADLs, IADLs, communication, a diagnosis of Alzheimer’s dementia, a diagnosis of dementia other than Alzheimer’s dementia, health instability) after stratifying by age.

While we do see value in more sophisticated statistical approaches, we have explicitly chosen, in this first paper, not to undertake multivariate analysis mainly because we see this paper as the first in a series of manuscripts using these types of data. We do plan on using multivariate techniques in future papers to enable us to better understand how sensory impairments may act as potential confounding variables and/or interact with cognitive impairment in influencing health-related outcomes. All analyses were performed using SAS Enterprise Guide (version 7.1)[65] and the reporting of the results was based on the STrengthening the Reporting of OBservational studies in Epidemiology (STROBE) guidelines.[66]

## Results

The results are presented in four main sections. We first describe the outcomes for each of the two cohorts separately, then compare the two groups, and end with the results from the age-stratified analysis.

### Home care clients

**Fig 1:**
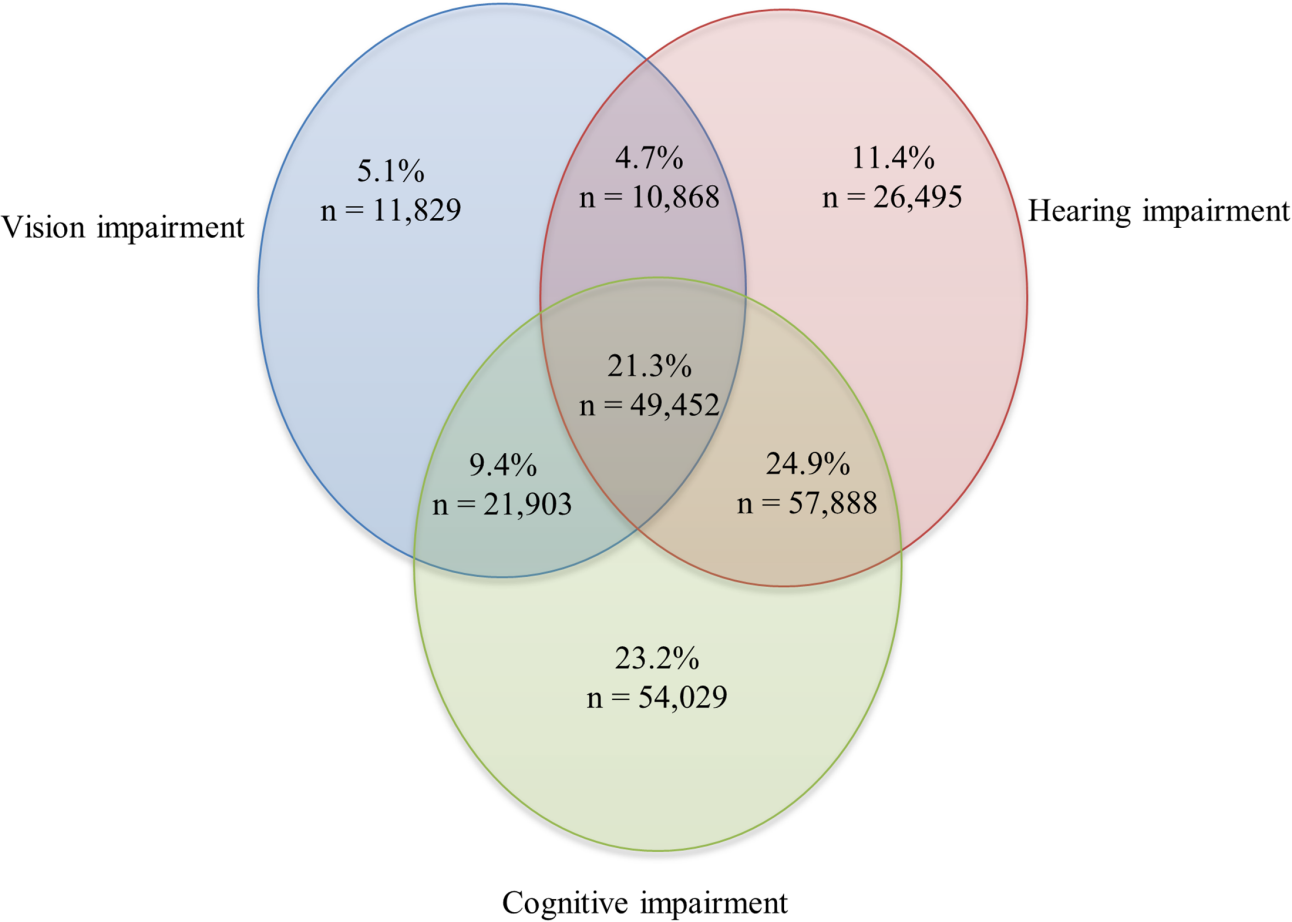
Distribution of home care clients with hearing and vision sensory and/or cognitive impairments. In this sample, 20.3% (n = 59,360) had none of these impairments.

The average age of home care clients in Ontario was 82.8 years (sd = 7.9); 61.1% were female; and over half (57.3%) were widowed, separated, or divorced. Most (79.6%; n = 232,464) of the clients exhibited some degree of sensory or cognitive impairment. Among the clients with sensory and/or cognitive impairment, the prevalence rates for *single impairments* ranged from 5.1% for VI alone, 11.4% for HI alone and 23.2% for CI alone. The rates for *combined* impairments ranged from 4.7% for DSI, 9.4% for CI+VI, and 24.9% for CI+HI. Clients experiencing all three impairments (i.e., CI+DSI) represented 21.3% of the sample (Fig 1).

Across the eight subgroups, clients with CI+DSI were the oldest, with 64.7% of clients in the 85+ age category having CI+DSI (Table 1). Compared to men, women had higher rates of impairment across all seven impairment subgroups. Marital status was not related to the presence of various types of impairments.

**Table 1:** Demographic characteristics of older home care clients in Ontario based on the presence of hearing and vision impairments and/or cognitive impairments.

Variables	No Impairment	Vision impairment (VI)	Hearing impairment (HI)	Dual sensory impairment (DSI)	Cognitive impairment (CI)	Cognitive + Sensory Impairment		
						CI + Vision impairment	CI + Hearing impairment	CI + DSI
	N = 59,360	N = 11,829	N = 26,495	N = 10,868	N = 54,029	N = 21,903	N = 57,888	N = 49,452
%								
**Age group (years)**								
65–74	37.5	28.4	15.6	11.0	19.3	18.5	7.2	6.5
75–84	43.0	41.4	39.8	31.5	46.6	42.5	33.8	28.8
85+	19.5	30.2	44.6	57.5	34.1	39.0	59.0	64.7
**Sex**								
Male	34.7	28.8	42.3	34.2	34.0	33.2	42.0	37.8
Female	65.3	71.2	57.7	65.8	66.0	66.8	58.0	62.2
**Client identifies self as First Nations, Métis or Inuit**	0.8	0.9	0.7	0.9	0.7	0.8	0.7	0.7
**Primary language**								
English	83.0	78.9	83.3	79.7	78.6	72.8	80.3	73.3
French	2.3	2.3	2.9	2.4	3.4	2.7	3.3	2.7
Other	14.7	18.8	13.8	17.9	18.0	24.5	16.4	24.0
**Marital status**								
Never married	4.7	5.3	3.6	4.0	4.8	4.7	3.4	3.6
Married	45.8	37.9	39.4	31.0	42.3	40.8	37.2	33.2
Widowed/separated/divorced	49.5	56.8	57.0	65.0	52.9	54.5	59.4	63.2
**Education**								
Less than high school	21.2	27.5	26.4	30.9	27.7	32.8	30.7	36.6
Some high school	17.9	18.2	19.4	19.9	17.2	17.1	19.2	17.6
High school or trade school	34.1	31.3	31.7	29.1	32.0	29.0	30.2	28.3
Post-secondary	26.8	23.0	22.5	20.1	22.1	21.1	19.9	17.5

Clients with CI+VI had the highest rate of procedural memory problems (56.1%; Table 2). Impaired skills for daily decision-making and worsening in decision-making over time were the most prevalent in clients with CI+DSI (64.9% and 42.1%, respectively). Finally, clients with CI+VI had the highest prevalence of symptoms of depression (28.2%).

**Table 2:** Cognition, mood, and behavioral patterns of older home care clients in Ontario based on the presence of hearing and vision impairments and/or cognitive impairments.

Variables	No Impairment	Vision impairment (VI)	Hearing impairment (HI)	Dual sensory impairment (DSI)	Cognitive impairment (CI)	Cognitive + Sensory Impairment		
						CI + Vision impairment	CI + Hearing impairment	CI + DSI
	N = 59,360	N = 11,829	N = 26,495	N = 10,868	N = 54,029	N = 21,903	N = 57,888	N = 49,452
%								
**Cognitive Performance Scale (CPS)**^a^								
Intact (0)	100.0	100.0	100.0	100.0	0.0	0.0	0.0	0.0
Borderline intact (1)	0.0	0.0	0.0	0.0	0.0	0.0	0.0	0.0
Mild impairment (2)	0.0	0.0	0.0	0.0	66.0	63.0	69.5	64.0
Moderate impairment (3)	0.0	0.0	0.0	0.0	20.3	19.1	18.8	19.2
Moderate/severe impairment (4)	0.0	0.0	0.0	0.0	2.8	3.7	2.7	3.7
Severe impairment (5)	0.0	0.0	0.0	0.0	9.5	10.6	8.1	10.0
Very severe impairment (6)	0.0	0.0	0.0	0.0	1.4	3.6	0.9	3.1
**Memory recall ability**								
Short-term memory problem	0.0	0.0	0.0	0.0	95.4	94.5	94.3	93.5
Procedural memory problem	0.4	0.7	0.6	0.8	55.0	56.1	51.6	55.2
**Cognitive skills for daily decision-making**								
Independent/modified independent (0–1)	100.0	100.0	100.0	100.0	37.0	36.0	39.1	35.1
Minimally to severely impaired (2–4)	0.0	0.0	0.0	0.0	63.0	64.0	60.9	64.9
**Worsening of decision-making as compared to 90 days ago**	0.5	0.7	0.5	0.8	42.1	41.2	41.9	42.1
**Client has become disoriented or agitated in the past 90 days**	0.5	0.5	0.6	0.6	10.0	10.0	9.5	10.0
**Wandering**	0.0	0.0	0.0	0.0	8.4	7.4	6.4	5.9
**Verbally abusive behavior**	0.4	0.5	0.5	0.7	8.3	8.3	7.3	7.4
**Physically abusive behavior**	0.0	0.0	0.0	0.1	2.6	2.5	1.9	2.2
**Socially inappropriate/disruptive behavioral symptoms**	0.1	0.1	0.1	0.1	4.7	4.4	4.0	4.1
**Depression Rating Scales (DRS)**								
No symptoms (0–2)	88.6	84.1	89.7	85.7	77.2	71.8	78.0	72.8
Symptoms (3–14)	11.4	15.9	10.3	14.3	22.8	28.2	22.0	27.2

^**a**^ score of 2+ on the CPS was used to determine the presence of CI

Clients with all three impairments were the most likely to experience difficulties in communication, even more so than individuals in the CI-alone group. For example, 38.0% of the CI+DSI group had moderate-to-severe difficulty understanding others, which was an increase of 12.9 percentage points over those with CI-alone (38.0% vs. 25.1%). Clients with all three impairments were also the most likely (28.4%) to experience a worsening of their communication (either expressive or receptive) in the previous three months. This represents an increase of 8.6 percentage points over the CI-alone group (19.8%).

The CI+DSI group also was the most likely, among the groups, to experience a number of negative health-related outcomes. For example, they were the most likely to report loneliness (17.0%), although the absolute difference between proportions was only 4% when compared with the CI-alone group (Table 3). Their primary caregivers were the most likely to report feelings of distress, anger, or depression (35.1%), which again, showed an absolute difference of 3.3% versus the CI only group (31.8%). This group also experienced the highest rates of impaired ADLs (57.5%), and difficulties with IADLs (82.0%) compared to all the other groups. The percent increase was 34.7% and 15.0% for ADLs and IADLs, respectively, when compared to individuals with only CI (Table 4). Compared to those with CI alone, individuals with CI+DSI also were more likely to experience declining vision over the past three months (17.9% vs. 2.0%) as well as having cataracts (20% vs. 9.3%).

**Table 3:** Communication ability, social functioning and caregiver status of home care clients in Ontario based on the presence of hearing and vision impairments and/or cognitive impairments.

Variables	No Impairment	Vision impairment (VI)	Hearing impairment (HI)	Dual sensory impairment (DSI)	Cognitive impairment (CI)	Cognitive + Sensory Impairment		
						CI + Vision impairment	CI + Hearing impairment	CI + DSI
	N = 59,360	N = 11,829	N = 26,495	N = 10,868	N = 54,029	N = 21,903	N = 57,888	N = 49,452
%								
**Hearing**								
Adequate	100.0	100.0	0.0	0.0	100.0	100.0	0.0	0.0
Mildly impaired	0.0	0.0	74.4	66.9	0.0	0.0	61.4	52.3
Moderately impaired	0.0	0.0	24.7	31.8	0.0	0.0	36.7	44.0
Severely impaired	0.0	0.0	0.9	1.3	0.0	0.0	1.9	3.7
**Vision**								
Adequate	100.0	0.0	100.0	0.0	100.0	0.0	100.0	0.0
Mildly impaired	0.0	70.9	0.0	67.2	0.0	68.6	0.0	63.7
Moderately impaired	0.0	17.3	0.0	19.7	0.0	19.4	0.0	21.5
Highly impaired	0.0	7.8	0.0	9.3	0.0	9.0	0.0	11.0
Severely impaired	0.0	4.0	0.0	3.8	0.0	3.0	0.0	3.8
**Vision decline in past 90 days**	2.2	20.0	3.1	21.0	2.0	17.2	2.7	17.9
**Ability to understand others (comprehension)**								
No difficulty	99.2	98.7	89.8	87.0	48.3	43.7	30.9	23.9
Mild difficulty	0.7	1.2	9.1	11.5	26.6	27.5	38.8	38.1
Moderate/severe difficulty	0.1	0.1	1.1	1.5	25.1	28.8	30.3	38.0
**Worsening in communication as compared to 90 days ago**	0.5	0.5	3.4	4.8	19.8	21.7	24.0	28.4
**Self-reported loneliness**	9.5	14.4	11.7	16.3	13.0	15.4	14.6	17.0
**Caregiver expresses feelings of distress, anger, or depression**	7.2	9.3	9.0	11.1	31.8	34.6	32.4	35.1

**Table 4:** Physical indicators of health and diagnoses of home care clients in Ontario based on the presence of hearing and vision sensory and/or cognitive impairments.

Variables	No Impairment	Vision impairment (VI)	Hearing impairment (HI)	Dual sensory impairment (DSI)	Cognitive impairment (CI)	Cognitive + Sensory Impairment		
						CI + Vision impairment	CI + Hearing impairment	CI + DSI
	N = 59,360	N = 11,829	N = 26,495	N = 10,868	N = 54,029	N = 21,903	N = 57,888	N = 49,452
%								
**Bladder incontinence**								
Continent	70.5	60.8	61.0	52.7	41.7	31.4	35.8	26.1
Any degree of incontinence	29.5	39.2	39.0	47.3	58.3	68.6	64.2	73.9
**Diagnoses**								
Hypertension	59.7	65.1	64.2	67.5	59.4	63.1	62.8	65.4
Diabetes	26.8	33.4	25.7	28.0	24.4	29.4	23.9	26.7
Coronary artery disease	23.6	27.6	29.6	31.2	22.8	24.7	28.8	29.6
Congestive heart failure	11.3	15.8	16.3	19.2	10.2	12.3	15.0	16.7
Stroke	9.6	14.2	11.4	15.0	19.6	26.1	20.1	24.3
Parkinsonism	2.1	2.8	2.1	2.3	6.0	8.5	5.0	6.3
Alzheimer’s dementia	0.0	0.0	0.0	0.0	20.1	15.5	15.0	11.8
Dementia other than Alzheimer’s dementia	0.0	0.0	0.0	0.0	37.1	34.1	35.7	33.5
Arthritis	49.2	55.2	58.2	63.7	44.9	48.5	54.0	56.6
Cataracts	10.9	23.1	13.2	22.4	9.3	19.0	10.9	20.0
Glaucoma	5.0	14.2	6.7	14.9	5.2	12.7	6.1	13.7
Any psychiatric diagnosis ^a^	9.0	10.6	8.7	8.7	19.0	18.8	16.6	16.5
Cancer	26.8	19.0	22.1	16.8	11.5	10.7	12.5	11.7
**Activities of Daily Living (ADL) Self-performance Hierarchy Scale**								
Independent/minor supervision (0–1)	83.1	76.7	81.1	74.3	57.3	45.5	55.3	42.5
Impairment (2–6)	16.9	23.3	18.9	25.7	42.7	54.5	44.7	57.5
**Instrumental Activities of Daily Living (IADL) Involvement Scale**								
None/minor difficulty (0–13)	83.1	72.4	78.2	65.2	28.7	21.1	26.2	18.0
Moderate/major difficulty (14–21)	16.9	27.6	21.8	34.8	71.3	78.9	73.8	82.0

^**a**^ Presence of any type of psychiatric diagnosis (e.g., depression, anxiety disorder, schizophrenia, paranoia)

As expected, the rates of Alzheimer’s dementia and other types of dementia were most common in home care clients with CI, with rates of 20.1% and 37.1%, respectively. Among those with CI+DSI, the rate of Alzheimer’s dementia was 11.8%, an absolute difference of 8.3% versus the CI group. In terms of those with a diagnosis of "other dementias", the CI group and the CI+DSI group were quite similar (absolute difference of 3.6%) (Table 4).

### Long-term care residents

**Fig 2:**
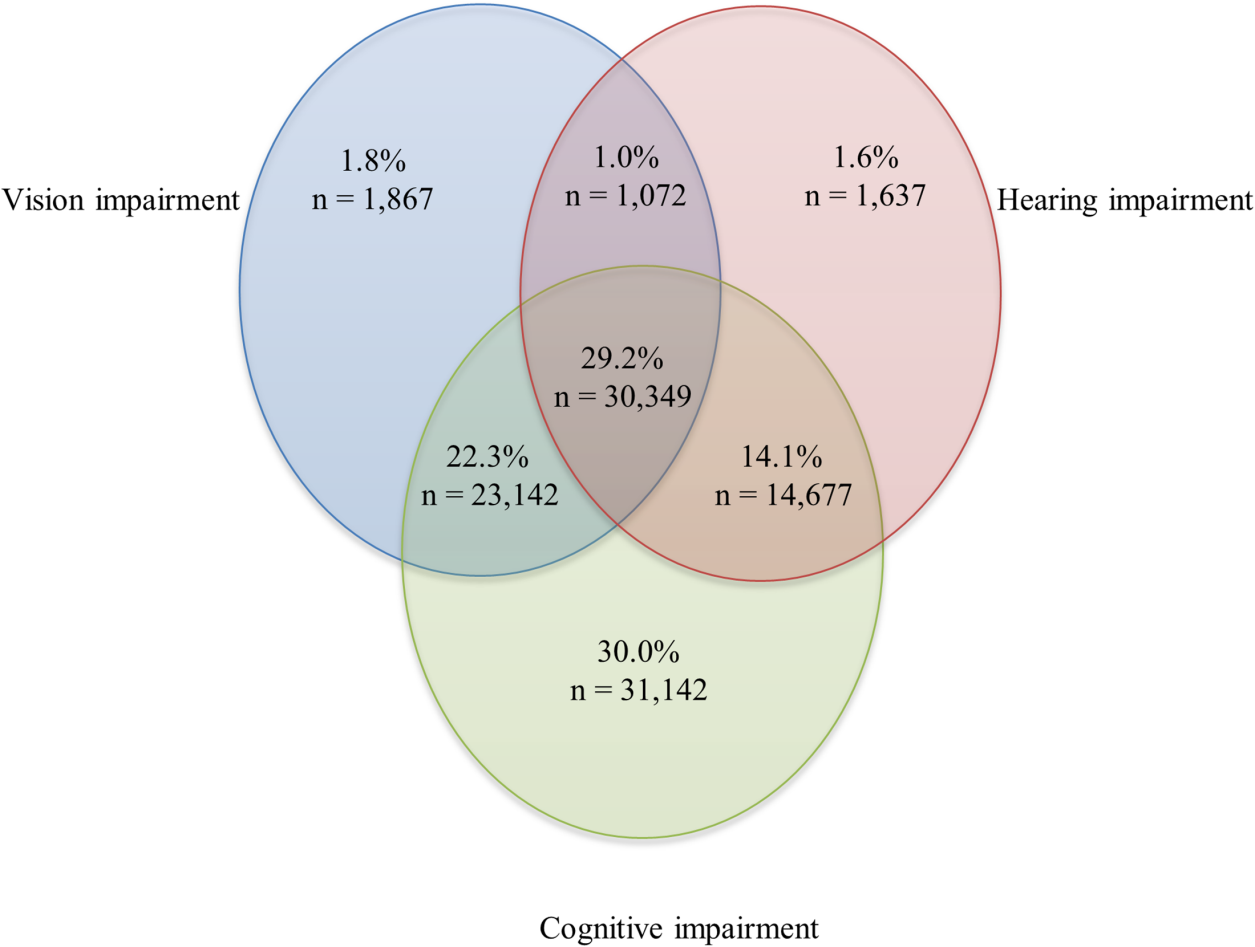
Distribution of long-term care residents with hearing and vision sensory and/or cognitive impairments. In this sample, 6.1% (n = 6,692) had none of these impairments.

LTC residents were older, on average, than home care clients (mean = 86.9 years of age; sd = 7.5); slightly more were female (69.5%); and a higher proportion were widowed, separated, or divorced (67.0%). Strikingly, the majority of residents (93.9%, n = 103,886) had some degree of sensory and/or cognitive impairment (Fig 2).

The prevalence of *single* impairments ranged from 1.6% for HI, 1.8% for VI, and 30.0% for CI. The rates for *combined* impairments ranged from 1.0% for DSI, 14.1% for CI+HI, 22.3% for CI+VI, and 29.2% for CI+DSI. What we see in the data from the LTC cohort is a slight shifting of the rates compared to the rates found for the home care setting, with lower rates for the single impairment groups and higher rates for the groups with impairments combined with CI. Since the overall prevalence of CI was higher in the LTC sample than in the home care sample (30.0% vs. 23.2%) and the impairment groups were mutually exclusive, there was a much higher proportion of LTC residents experiencing some combination of two or more impairments.

LTC residents with DSI were the oldest, accounting for 81.6% of those in the 85+ age group (Table 5). Female residents had higher rates across all of the single and combined impairments than male residents. Being widowed, separated or divorced was the most common marital status and clients with DSI had the highest rate compared to other impairment groups (74.6%).

**Table 5:** Demographic characteristics of older adults residing in a long-term care facility in Ontario based on the presence of hearing and vision impairments and/or cognitive impairments.

Variables	No Impairment	Vision impairment (VI)	Hearing impairment (HI)	Dual sensory impairment (DSI)	Cognitive impairment (CI)	Cognitive + Sensory Impairment		
						CI + Vision impairment	CI + Hearing impairment	CI + DSI
	N = 6,692	N = 1,867	N = 1,637	N = 1,072	N = 31,142	N = 23,142	N = 14,677	N = 30,349
%								
**Age group**								
65–74	17.1	12.9	4.6	3.6	10.5	8.9	3.6	3.6
75–84	34.6	29.3	21.6	14.8	36.1	32.7	22.7	20.6
85+	48.3	57.8	73.8	81.6	53.4	58.4	73.7	75.8
**Sex**								
Male	31.7	29.7	35.1	29.9	29.5	26.5	33.0	28.0
Female	68.2	70.1	64.9	69.9	70.3	73.4	66.8	71.8
Other	0.1	0.2	0.0	0.2	0.2	0.1	0.2	0.2
**Primary language**								
English	84.7	83.6	84.6	84.5	80.2	78.3	83.1	79.9
French	3.6	3.6	5.3	2.4	4.1	3.5	4.8	3.6
Other	11.7	12.8	10.1	13.1	15.7	18.2	12.1	16.5
**Marital status**								
Never married	9.4	8.4	8.3	6.5	7.4	7.0	6.5	6.0
Married	24.8	23.5	22.8	18.9	29.3	28.4	25.3	23.7
Widowed/separated/divorced	65.8	68.1	68.9	74.6	63.3	64.6	68.2	70.3
**Education**								
Less than high school	29.3	32.0	31.7	31.9	31.9	34.7	34.2	37.1
Some high school	20.5	20.7	17.9	16.1	17.0	16.7	17.5	16.2
High school or trade school	32.9	31.1	33.9	36.0	33.0	31.8	31.1	30.5
Post-secondary	17.3	16.2	16.5	16.0	18.1	16.8	17.2	16.2

Similar to the home care sample, residents with all three impairments (CI + DSI) were more likely to experience a greater number of negative health-related outcomes when compared to all seven other subgroups. For example, this group displayed the highest rates of memory problems, with 97.4% experiencing short-term memory problems and 85.5% experiencing problems with long-term memory. The rate in the CI group was very similar to this, with an absolute difference of less than 10% for both types of memory problems. (Table 6). The group with all three impairments also showed the highest rates on the six indicators of delirium, although the CI-alone group was very similar. The highest prevalence of depressive symptoms was found among residents with CI+HI (39.8%), followed closely by the CI+DSI group (37.6%) and then the CI-alone group (34.2%).

**Table 6:** Cognition, mood, and behavioral patterns of older adults residing in a long-term care facility in Ontario based on the presence of hearing and vision impairments and/or cognitive impairments.

Variables	No Impairment	Vision impairment (VI)	Hearing impairment (HI)	Dual sensory impairment (DSI)	Cognitive impairment (CI)^a^	Cognitive + Sensory Impairment		
						CI + Vision impairment	CI + Hearing impairment	CI + DSI
	N = 6,692	N = 1,867	N = 1,637	N = 1,072	N = 31,142	N = 23,142	N = 14,677	N = 30,349
%								
**Problems with short-term memory**	0.0	0.0	0.0	0.0	96.2	97.2	95.8	97.4
**Problems with long-term memory**	0.6	1.1	0.6	0.9	78.5	85.0	74.6	85.5
**Cognitive skills for daily decision-making**								
Independent/modified independent (0–1)	100.0	100.0	100.0	100.0	22.6	13.7	26.0	13.2
Moderate/severely impaired (2–4)	0.0	0.0	0.0	0.0	77.4	86.3	74.0	86.8
**Indicators of delirium**								
Easily distracted	7.3	9.1	10.3	9.8	53.0	54.4	55.7	58.2
Altered perceptions	2.6	3.9	3.7	5.5	37.2	41.7	40.7	48.3
Disorganized speech	2.1	3.1	3.4	3.3	35.5	41.6	36.7	45.9
Restlessness	3.6	5.8	3.7	4.9	33.1	37.9	34.1	42.5
Lethargy	9.1	10.6	11.6	14.1	30.7	36.9	34.5	43.5
Varying mental function	7.9	7.9	10.0	10.6	43.7	43.5	46.8	48.5
**Cognitive Performance Scale (CPS)**								
Intact (0)	100.0	100.0	100.0	100.0	0.0	0.0	0.0	0.0
Borderline intact (1)	0.0	0.0	0.0	0.0	0.0	0.0	0.0	0.0
Mild impairment (2)	0.0	0.0	0.0	0.0	21.5	12.9	25.1	12.6
Moderate impairment (3)	0.0	0.0	0.0	0.0	44.6	34.0	47.0	34.2
Moderate/severe impairment (4)	0.0	0.0	0.0	0.0	8.4	9.8	8.7	11.5
Severe impairment (5)	0.0	0.0	0.0	0.0	13.7	17.7	12.1	17.9
Very severe impairment (6)	0.0	0.0	0.0	0.0	11.8	25.6	7.1	23.8
**Depression Rating Scale (DRS)**								
No symptoms (0–2)	78.0	75.6	74.5	75.1	65.8	67.2	60.2	62.4
Symptoms (3–14)	22.0	24.4	25.5	24.9	34.2	32.8	39.8	37.6

^**a**^ score of 2+ on the CPS was used to determine the presence of CI

LTC residents with CI+DSI were more likely than the other groups to have communication problems and reduced social participation (Table 7). A total of 49.2% of residents had moderate/severe difficulty in understanding others, which represents a 77.6% increase in the proportion versus the CI-alone group (27.7%), indicating a marked increase in communication difficulties in the presence of multiple sensory impairments combined with CI.

**Table 7:** Communication ability and social functioning of older adults residing in a long-term care facility in Ontario based on the presence of vision and hearing impairments and/or cognitive impairments.

Variables	No Impairment	Vision impairment (VI)	Hearing impairment (HI)	Dual sensory impairment (DSI)	Cognitive impairment (CI)	Cognitive + Sensory Impairment		
						CI + Vision impairment	CI + Hearing impairment	CI + DSI
	N = 6,692	N = 1,867	N = 1,637	N = 1,072	N = 31,142	N = 23,142	N = 14,677	N = 30,349
%								
**Hearing**								
Adequate	100.0	100.0	0.0	0.0	100.0	100.0	0.0	0.0
Mildly impaired	0.0	0.0	72.2	66.7	0.0	0.0	68.2	59.2
Moderately impaired	0.0	0.0	24.9	29.1	0.0	0.0	27.8	33.7
Severely impaired	0.0	0.0	2.9	4.2	0.0	0.0	4.0	7.1
**Vision**								
Adequate	100.0	0.0	100.0	0.0	100.0	0.0	100.0	0.0
Mildly impaired	0.0	67.8	0.0	63.5	0.0	59.2	0.0	54.1
Moderately impaired	0.0	16.3	0.0	22.1	0.0	17.5	0.0	21.6
Highly impaired	0.0	8.1	0.0	9.3	0.0	19.2	0.0	18.8
Severely impaired	0.0	7.8	0.0	5.1	0.0	4.1	0.0	5.5
**Visual appliance** (e.g., magnifying glass)	74.2	73.3	83.9	78.3	51.9	49.3	64.5	54.1
**Communication devices**								
Hearing aids present and used regularly	10.9	10.0	39.9	40.6	3.4	2.3	18.8	12.1
Hearing aids preset and not used regularly	1.3	1.6	12.7	10.4	0.8	0.7	10.9	8.9
Other receptive communication techniques (e.g., lip reading)	0.1	0.1	2.6	2.2	0.2	0.2	2.1	2.2
**Ability to understand others (comprehension)**								
No difficulty	99.2	99.0	88.5	85.3	35.2	22.1	19.9	9.9
Mild difficulty	0.8	1.0	11.4	14.4	37.1	33.7	54.6	40.9
Moderate/severe difficulty	0.0	0.0	0.1	0.3	27.7	44.2	25.5	49.2
**Worsening in communication as compared to 90 days ago**	0.2	0.3	1.9	1.4	4.7	6.3	6.8	9.0
**Sense of initiative or involvement**								
At ease interacting with others	93.5	92.4	94.7	91.1	75.8	66.1	78.3	65.0
At ease doing planned activities	75.4	72.7	73.9	66.6	56.4	46.7	57.4	44.3
At ease doing self-initiated activities	88.9	84.0	91.5	85.5	46.8	32.1	54.4	32.5
Establishing own goals	72.4	68.7	76.4	70.7	31.3	22.1	37.3	23.2
Pursues involvement in life of facility	49.6	44.7	49.4	42.3	22.9	16.2	22.9	14.0
Accepts invitation in groups activities	32.0	31.8	32.8	29.6	34.7	29.8	32.9	26.9

The highest rate of deterioration in communication (over the previous three months) also was seen among those with CI+DSI (9.0%). Further, residents with CI+DSI experienced the *lowest* rates, based on the raw proportions, on five of six measures associated with involvement in social activities, including interacting with others, participating in planned or self-initiated activities, and accepting invitations to join group activities. Even in the presence of multiple impairments, residents with CI+DSI showed a relatively low rate for the use of hearing aids (8.9%) and visual support aids (54.1%).

LTC residents with CI+DSI showed the highest rates of bladder incontinence (92.7%), which was very similar to the CI group (87.0%; Table 8). Alzheimer’s dementia was most prevalent among residents with CI+VI (26.8%), and other types of dementia were most common, across the various subgroups, among those with CI+DSI (60.2%). Nearly all residents with CI+VI had impaired ADLs (97.3%), which also was true for the group with CI+DSI (97.1%). ADL impairment was more prevalent among residents with CI+DSI compared to those residents experiencing no impairments (67.1%), representing a 30% difference in the two proportions.

**Table 8:** Physical indicators of health and diagnoses of older adults residing in a long-term care facility in Ontario based on the presence of vision and hearing impairments and/or cognitive impairments.

Variables	No Impairment	Vision impairment (VI)	Hearing impairment (HI)	Dual sensory impairment (DSI)	Cognitive impairment (CI)	Cognitive + Sensory Impairment		
						CI + Vision impairment	CI + Hearing impairment	CI + DSI
	N = 6,692	N = 1,867	N = 1,637	N = 1,072	N = 31,142	N = 23,142	N = 14,677	N = 30,349
%								
**Bladder incontinence**								
Continent	40.7	37.9	39.5	36.5	13.0	7.4	13.3	7.3
Any degree of incontinence	59.3	62.1	60.5	63.5	87.0	92.6	86.7	92.7
**Diagnoses**								
Hypertension	65.3	65.4	66.2	68.3	59.7	58.0	63.3	59.0
Diabetes	31.5	33.9	27.0	24.7	25.9	26.3	24.5	23.4
Congestive heart failure	20.3	19.1	23.3	23.1	11.6	11.1	15.8	14.4
Stroke	20.6	19.3	16.8	18.3	22.7	23.9	21.5	22.5
Parkinsonism	6.4	5.4	5.3	4.2	8.0	8.9	6.7	7.6
Alzheimer’s dementia	0.0	0.0	0.0	0.0	24.7	26.8	20.1	24.2
Dementia other than Alzheimer’s dementia	0.0	0.0	0.0	0.0	58.5	58.8	58.9	60.2
Arthritis	48.8	50.7	55.7	54.9	39.5	40.3	48.6	46.5
Cataracts	11.1	22.2	13.2	21.5	8.8	16.3	10.6	17.9
Glaucoma	7.1	17.9	9.5	19.1	5.3	11.1	6.6	12.5
Diabetic retinopathy	0.7	3.6	0.7	2.0	0.3	1.0	0.3	0.8
Macular degeneration	4.3	22.0	6.0	28.2	2.8	9.0	4.3	13.2
Cancer	10.6	9.6	13.3	13.1	8.5	8.4	10.7	9.9
**Number of chronic co-morbid conditions**								
0–1	3.5	3.4	3.2	2.0	2.6	1.8	1.6	1.6
2	7.0	5.1	5.3	4.9	6.8	5.7	4.8	4.5
3+	89.5	91.5	91.5	93.1	90.6	92.5	93.6	93.9
**Activities of Daily Living (ADL) Self-performance Hierarchy Scale**								
Independent/minor supervision (0–1)	32.9	26.8	33.4	27.8	6.9	2.7	7.3	2.9
Impairment (2–6)	67.1	73.2	66.6	72.2	93.1	97.3	92.7	97.1

### Comparison of those living in LTC and those receiving home care

Comparing the overall highest rates for those living in LTC to those receiving home care services, regardless of level or combination of sensory and cognitive impairment, LTC residents experienced a 33.7% increase in the proportion with difficulties in daily decision-making (86.8% vs. 64.9%). The rate of moderate/severe difficulties with receptive communication was also 29.5% higher in LTC versus home care clients (49.2% vs. 38.0%). ADL impairment showed some of the largest overall differences between the two cohorts, with LTC residents displaying a 69.2% increased rate of ADL impairments (97.3% vs. 57.5%).

### Age stratification

**Fig 3:**
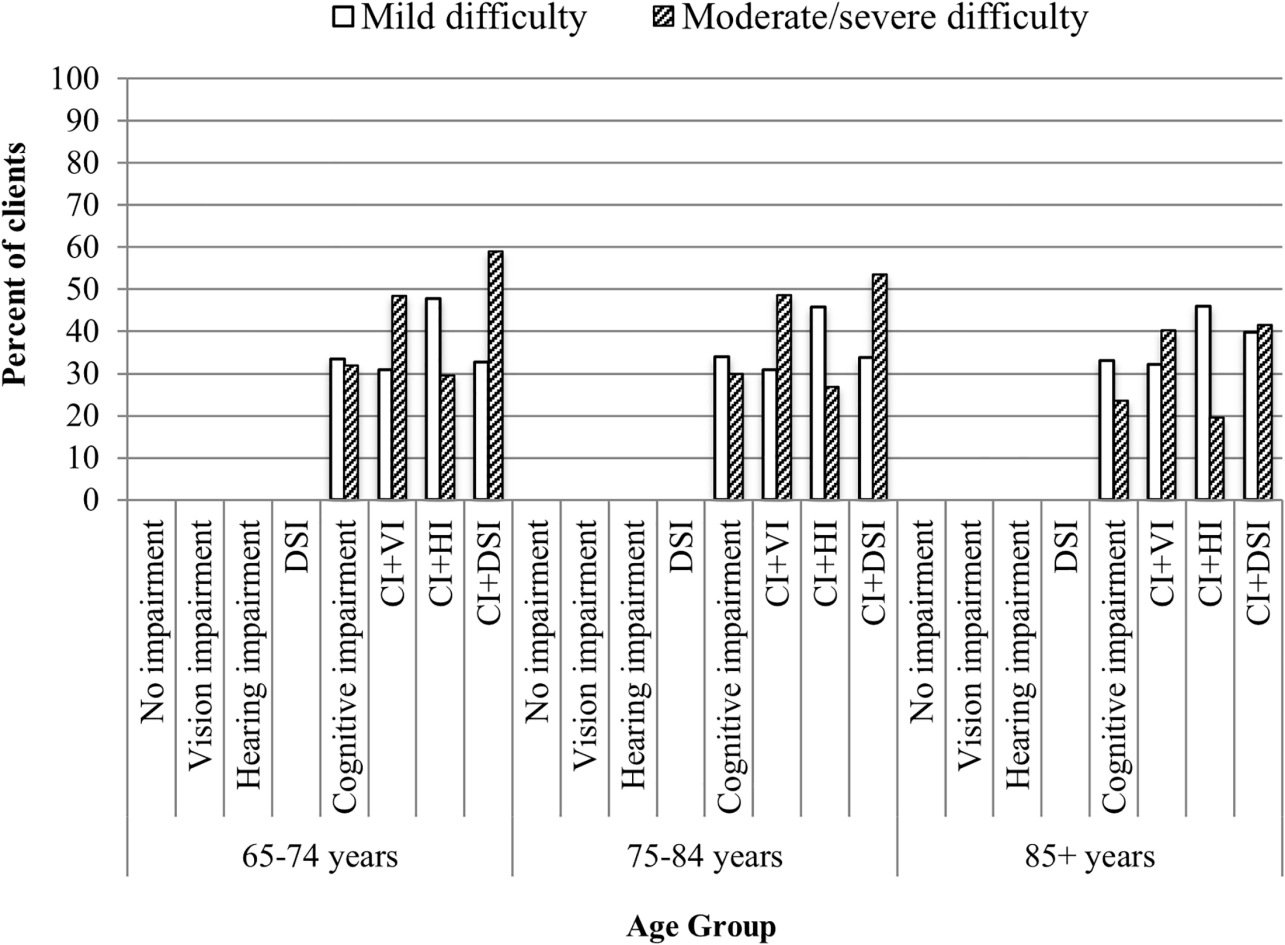
Age stratification for degree of difficulty in being understood in long-term care residents with hearing and vision sensory and/or cognitive impairments.

**Fig 4:**
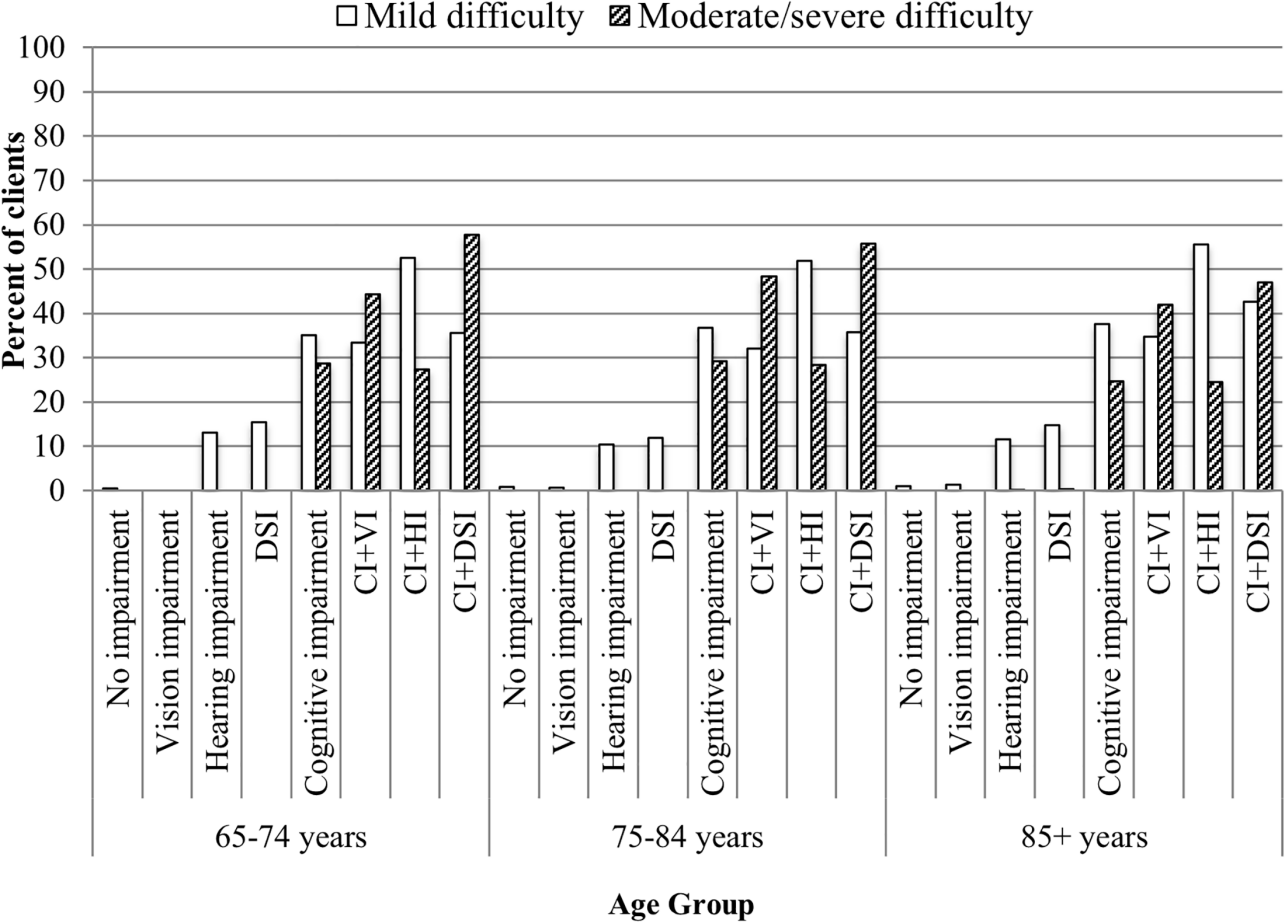
Age stratification for degree of difficulty in understanding others in long-term care residents with hearing and vision sensory and/or cognitive impairments.

The age-stratified analysis, conducted separately for home care clients and LTC residents, showed that the relationships remained relatively consistent and typically varied by less than 10% across the three age categories in both samples. An interesting exception to this pattern included items related to both expressive and receptive communication in the LTC sample. Residents with CI+DSI showed the greatest variation by age. For instance, the prevalence rates for moderate/severe difficulty being understood among residents aged 65–74 was 59.0%, which decreased to 53.6% for residents 75–84 years, and further decreased to 41.5% in the oldest age group (85+). What also was apparent was the fact that the proportion of residents who showed moderate/severe difficulty in expressing themselves was *lower* in the CI+HI group relative to the CI+VI group (Fig 3). This pattern also held true for receptive communication, perhaps suggesting that the HI group with no visual impairments are able to utilize body language, facial expressions or other positive adaptive strategies when interacting with others (Fig 4).

## Discussion

To our knowledge, this is the first study on the relationships among combined sensory and cognitive impairments and the associations with several important health outcomes in older adults living in LTC or receiving home care. We found high rates of combined vision, hearing, and cognitive impairments representing one-fifth and just under one-third of home care clients and LTC residents, respectively. Those with multiple impairments are a particularly vulnerable group with unique needs and challenges. Across several important outcomes known to be associated with older adults’ health (e.g., functional independence, skills for daily decision-making and communication), the rates were higher among individuals with CI+DSI versus those with CI-alone. Although not surprising, this is an important new finding that demonstrates of the extent of the effect of combined sensory and cognitive impairments.

The proportion of individuals with DSI in our study was very similar to that found in a study of home care clients in 10 European countries,[13, 22] that also used data from the RAI-HC, and in a Japanese study of older adults eligible for LTC insurance.[41] However, the proportion with DSI in the current study was higher than in another recent European study, which reported rate of 6%, likely due to the fact that the latter was based strictly on self-report.[22] In our study, the completion of the interRAI tools by trained professionals involves a combination of self-report and the assessed level of impairment as judged by the individual assessor. The assessor can include information from informal care providers and standard medical tests, when they are available. It is crucial to involve older adult clients in the assessment of their sensory abilities because gold standard clinical measures are not necessarily the ideal way to understand comprehensively the influences of sensory challenges on everyday functioning.[67, 68]

Individuals with all three impairments (i.e., CI+DSI) typically had the highest rates of functional impairments, difficulties with communication and a worsening in their communication abilities over time, based on items within the assessment tools, when compared with the other groups. For example, they displayed the highest rates of difficulty with both expressive and receptive communication. When compared to participants who only had CI, participants with CI+DSI were even more likely to experience difficulty understanding others. Our findings show that there is an additive effect of DSI on communication problems above and beyond the presence of CI alone. The current study is the first to show such results for communication since other published papers that explored the combination of sensory and cognitive impairments did not include communication-related outcomes.[40–44] Our age-stratified analysis showed that having CI+VI increased the prevalence of difficulty understanding others compared to those with CI+HI, regardless of age. This finding is due likely to the fact that visual-speech cues improve the ability of older adults to recognize and understand speech even in the presence of hearing and vision impairments.[69]

Caregivers of home care clients who had CI and multiple sensory impairments were much more likely to report feeling stressed, angry or depressed as a result of their caregiving role, versus caregivers of those who had CI alone. There is clear evidence that caring for someone with dementia increases the likelihood of experiencing the multiple dimensions of caregiver burden [70–72]. However, to date only a few published studies report on the influences of sensory impairments on caregiver burden.[73–78] We were unable to explore this relationship in the LTC cohort since the question on caregiver status is not part of the interRAI tool used in LTC settings. Although not the primary focus of the current project, future research examining the influence of sensory and/or cognitive impairment on caregiver burden by family members and professionals is warranted given that both issues are on the rise and both result in changes in communication that create challenges for both clients and their caregivers and may result in family members exiting the caregiver role and also affect the quality of care provided by personal service workers in home care or staff in LTC.

The presence of DSI combined with CI increased the likelihood of limited independence on IADLs compared to those with CI-alone. One other study also examined DSI as a risk factor for functional limitations, but did not find that those with DSI had a greater risk compared with individuals with a single sensory impairment. The investigators, however, did not explore the additive effect of CI+DSI as a risk factor for functional difficulties.[79]

Although the interRAI assessments provide a rich source of information about older adults in these two settings, we were limited to the data elements available within each of these tools. As such, we were unable to determine when the sensory or cognitive impairment arose because the date of onset is not recorded on these assessments. Our two samples include a mix of individuals with a new impairment as well as those with a long-standing impairment. We also had no information about the frequency of the use of devices to assist with a person’s hearing or vision in the home care sample. Moreover, we have limited information on the use of such devices in the LTC sample. The assessment instruments, although not purely based on self-report, are nevertheless subjective in nature. They capture the functional aspects of hearing, vision and cognition, but are unable to quantify these impairments in an objective manner.

The current study is cross-sectional and descriptive in nature and meant to be foundational for future research in this area. Thus, we are unable to determine causal linkages between the various types of impairments and important health-related outcomes. However, these types of more sophisticated analyses (e.g., multivariate survival analysis) will be undertaken by our group in the future since many individuals within these two settings will have multiple assessments to allow for tracking of cohorts over time and the use of multivariate techniques will provide further insight into these complex relationships.

The results of this study are vital to advancing our understanding of how best to enhance the health and quality of life of older adults with sensory impairment(s) and/or cognitive impairment and their caregivers. In order to meet person-centered or family-centered goals of care for older adults, adequate two-way communication needs to be established between older adults and their informal caregivers and professional health care providers. In the presence of hearing, vision, and cognitive impairments, communication becomes particularly challenging which, in turn, can negatively affect the quality of care provided.[80, 81] Furthermore, longstanding data show that age-related hearing impairment will affect most older adults. Hearing impairment in the context of aging is an important risk factor in the development of cognitive challenges.[82] Screening for hearing impairments may increase the likelihood that older adults will be prescribed and use a hearing aid,[83] and yet effective sensory interventions (e.g., assistive technologies, vision/hearing rehabilitation) and contact with health care professionals are often under-utilized strategies.[84–87] Part of the issue may be a lack of understanding of how best to screen for hearing and vision impairment in dementia,[88] and once identified, how to prescribe and fit appropriate devices in this population and to provide other rehabilitative services for the individual and their caregivers. Given the effect of cognitive impairment and sensory impairment on health-related outcomes in a large proportion of this study cohort, the potential to improve quality of life and effective care delivery by identifying effective screening and interventions is significant.

What is clearly lacking is a broad public health perspective that recognizes the significance of screening for hearing and vision sensory impairments both alone and in combination with cognitive challenges, and the potentially negative outcomes associated with failing to do so. It is imperative for all health care professionals who work with older adults to recognize the potential of hearing, vision, and cognitive impairments in those for whom they provide care, and for them to ensure that basic screening takes place, similar to what is included in the interRAI tools. Screening for sensory and cognitive impairments is an integral part of a comprehensive geriatric assessment[89] and represents an important first step in identifying strengths and weaknesses so that appropriate referrals and interventions can be put in place. Hearing, vision, and cognitive screenings and implementing appropriate interventions will provide older adults and their caregivers with valuable information to help them understand and enhance their current communication abilities, to optimize their health status and to help them make informed decisions that could readily improve their quality of life.

## Supporting information

S1 File Other characteristics of home care clients and long-term care residents in Ontario based on the presence of hearing and vision sensory and/or cognitive impairments. (DOCX)
