# Supplementary material for: Combined impairments in vision, hearing and cognition are associated with greater levels of functional and communication difficulties than cognitive impairment alone: Analysis of interRAI data for home care and long-term care recipients in Ontario
Source: PLoS One. 2018 Feb 15;13(2):e0192971. doi: 10.1371/journal.pone.0192971 (PMC5814012; doi:10.1371/journal.pone.0192971)
Supplement: S1 File — (DOCX) [file pone.0192971.s001.docx]

Table A: Other characteristics of home care clients in Ontario based on the presence of hearing and vision sensory and/or cognitive impairments

| **Variables** | **No Impairment** | **Vision impairment (VI)** | **Hearing impairment (HI)** | **Dual sensory impairment (DSI)** | **Cognitive impairment (CI)** | **Cognitive + Sensory Impairment** | | |
| --- | --- | --- | --- | --- | --- | --- | --- | --- |
|  |  |  |  |  |  | **CI + Vision impairment** | **CI + Hearing impairment** | **CI + DSI** |
|  | **N=59,360** | **N=11,829** | **N=26,495** | **N=10,868** | **N=54,029** | **N=21,903** | **N=57,888** | **N=49,452** |
| % | | | | | | | | |
|  |  |  |  |  |  |  |  |  |
| **Client and/or primary caregiver feels that client would be better off in another living environment** | | | | | | | | |
| No | 91.3 | 86.3 | 86.9 | 82.1 | 56.8 | 56.5 | 55.0 | 53.3 |
| Client only | 2.2 | 3.2 | 2.5 | 3.0 | 1.6 | 1.6 | 1.4 | 1.6 |
| Caregiver only | 1.6 | 2.5 | 2.6 | 3.7 | 26.7 | 24.6 | 25.4 | 24.5 |
| Client and caregiver | 4.9 | 8.0 | 8.0 | 11.2 | 14.9 | 17.3 | 18.2 | 20.6 |
| **Experienced** **an overnight hospital stay** | 43.1 | 37.1 | 39.3 | 33.9 | 27.6 | 28.0 | 28.7 | 28.5 |
| **Experienced** **an emergency room visit** | 22.7 | 21.4 | 23.2 | 22.1 | 20.9 | 21.0 | 22.2 | 22.3 |
| **Receipt of formal care ^a^** | | | | | | | | |
| Home health aide | 46.5 | 57.8 | 55.0 | 64.2 | 62.7 | 71.1 | 69.4 | 75.5 |
| Visiting nurse | 38.0 | 30.8 | 32.7 | 27.4 | 24.4 | 25.0 | 26.7 | 27.0 |
| Homemaking service | 24.0 | 30.8 | 31.5 | 37.3 | 34.7 | 36.4 | 40.6 | 40.9 |
| Meals | 5.9 | 8.5 | 8.8 | 11.9 | 13.8 | 14.1 | 16.1 | 16.4 |
| Physical therapy | 15.0 | 12.5 | 13.7 | 12.1 | 10.6 | 10.6 | 11.4 | 11.1 |
| Occupational therapy | 15.5 | 14.7 | 14.3 | 12.7 | 13.4 | 13.2 | 12.3 | 12.7 |
| Day care or hospital care | 0.9 | 1.1 | 0.9 | 1.0 | 5.9 | 4.9 | 4.5 | 3.6 |
| **Change in social activities** | | | | | | | | |
| No decline | 55.4 | 57.7 | 58.8 | 59.4 | 58.8 | 55.5 | 57.7 | 53.9 |
| Decline, not distressed | 29.5 | 26.2 | 27.1 | 26.3 | 30.7 | 32.2 | 32.1 | 34.0 |
| Decline, distressed | 15.1 | 16.1 | 14.1 | 14.3 | 10.5 | 12.3 | 10.2 | 12.1 |
| **Number of chronic co-morbid conditions** | | | | | | | | |
| 0-1 | 14.6 | 8.5 | 9.8 | 6.9 | 9.0 | 6.2 | 6.5 | 5.2 |
| 2 | 21.1 | 15.7 | 17.3 | 13.7 | 17.0 | 13.2 | 13.8 | 11.4 |
| 3+ | 64.3 | 75.8 | 72.9 | 79.4 | 74.0 | 80.6 | 79.7 | 83.4 |
| **Change in Health, End-stage disease, Signs and Symptoms Scale (CHESS)** | | | | | | | | |
| None/mild instability (0-1) | 64.9 | 62.3 | 60.2 | 57.9 | 50.9 | 46.8 | 45.6 | 42.4 |
| Moderate/severe instability (2-5) | 35.1 | 37.7 | 39.8 | 42.1 | 49.1 | 53.2 | 54.4 | 57.6 |
| **Pain Scale** | | | | | | | | |
| No pain/less than daily (0-1) | 39.0 | 35.8 | 37.0 | 34.6 | 57.9 | 52.0 | 51.4 | 46.3 |
| Daily/severe pain (2-3) | 61.0 | 64.2 | 63.0 | 65.4 | 42.1 | 48.0 | 48.6 | 53.7 |

**^a^** Receipt of any level of the service within the previous 7 days. Other formal services captured on RAI-HC (e.g., meals, volunteer service, physical therapy, occupational therapy, speech therapy, day care/day hospital, and social worker) all had rates of less than 5% across each of the single and combined impairment groups and were therefore not included in the table.

Table B: Other characteristics of long-term care residents in Ontario based on the presence of hearing and vision sensory and/or cognitive impairments

| **Variables** | **No Impairment** | **Vision impairment (VI)** | **Hearing impairment (HI)** | **Dual sensory impairment (DSI)** | **Cognitive impairment (CI)** | **Cognitive + Sensory Impairment** | | |
| --- | --- | --- | --- | --- | --- | --- | --- | --- |
|  |  |  |  |  |  | **CI + Vision impairment** | **CI + Hearing impairment** | **CI + DSI** |
|  | **N=6,692** | **N=1,867** | **N=1,637** | **N=1,072** | **N=31,142** | **N=23,142** | **N=14,677** | **N=30,349** |
| % | | | | | | | | |
| **Deteriorated cognitive status** | 1.6 | 2.2 | 3.4 | 3.1 | 8.7 | 8.9 | 11.3 | 12.0 |
| **Behavioral symptoms** |  |  |  |  |  |  |  |  |
| Wandering | 0.7 | 0.5 | 0.5 | 0.8 | 20.8 | 16.5 | 19.7 | 16.8 |
| Verbally abusive behaviour | 6.8 | 7.8 | 5.7 | 6.1 | 22.1 | 21.2 | 21.8 | 22.5 |
| Physically abusive behaviour | 0.7 | 0.7 | 0.5 | 0.8 | 15.4 | 17.6 | 14.5 | 18.5 |
| Socially inappropriate behaviour | 4.2 | 4.8 | 4.1 | 2.5 | 20.4 | 21.5 | 21.0 | 23.5 |
| Resists care | 9.7 | 11.6 | 10.7 | 11.1 | 44.5 | 48.5 | 43.9 | 49.5 |
| **Change in Health, End-stage disease, Signs and Symptoms Scale (CHESS)** | | | | | | | | |
| None/mild instability (0-1) | 82.5 | 82.2 | 78.1 | 77.9 | 78.1 | 75.6 | 71.3 | 70.0 |
| Moderate/severe instability (2-5) | 17.5 | 17.8 | 21.9 | 22.1 | 21.9 | 24.4 | 28.7 | 30.0 |
| **Pain Scale** | | | | | | | | |
| No pain/less than daily (0-1) | 79.7 | 76.9 | 77.9 | 78.3 | 89.2 | 88.5 | 86.4 | 86.0 |
| Daily/severe pain (2-3) | 20.3 | 23.1 | 22.1 | 21.7 | 10.8 | 11.5 | 13.6 | 14.0 |
